# Morgagni Hernia presenting as Obstructive Jaundice

**Published:** 2015-09-01

**Authors:** Prince Raj, Yogesh Kumar Sarin

**Affiliations:** Department of Pediatric Surgery Maulana Azad Medical College, and associated LokNayak Hospital, New Delhi

**Dear Sir,**

Obstructive jaundice in an infant has varied etiology but Morgagni hernia (MH) leading to it is extremely rare. We hereby report a case of 10-month male child with obstructive jaundice.

A 10-month-old male infant presented with complaints of yellowish discoloration of eyes and urine for last 25 days. He was a product of a non-consanguineous marriage, full term born at home through normal vaginal delivery. The birth weight was 2.5 kg. There was a history of delayed cry. In view of the developmental delay noted, he was being treated in Child Developmental Clinic. He was referred to us for the management of incidentally diagnosed obstructive jaundice; the infant was otherwise asymptomatic. On physical examination, the baby had facial features of Down syndrome with gross developmental delay and yellowish discoloration of sclera. Pectus carinatum was also noted.

Biochemical study revealed serum bilirubin of 8 mg% with direct bilirubin of 2.8 mg%. Ultrasound abdomen was not helpful and x ray abdomen and chest revealed bowel loops in thorax. Following this a lateral chest x ray was done which showed the anterior position of bowel in right hemithorax. MRCP was done which revealed dilatation of common bile duct and intra hepatic biliary radical (Fig. 1).

**Figure:**
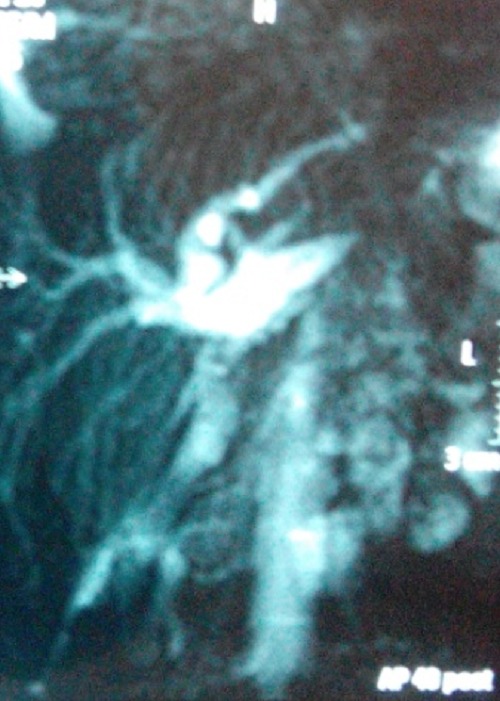
Figure 1: MRCP showing dilated intrahepatic biliary radicals and common bile duct with tapering and upward traction of distal bile duct.

Exploratory laparotomy through subcostal Mercedes-Benz incision confirmed MH with sac having transverse colon and large part of small intestine including duodenum as contents. Gall bladder and common bile duct were dilated with the traction on its distal part. The sac was firmly adherent to the pericardium. Primary repair of the defect was done. Child had a stormy post operative course. Though his serum bilirubin showed a decreasing trend, he continued to be febrile. In spite of the intensive management, he died on 6thpostoperative day on account of sepsis related meningitis and hepatic failure.

MH has high incidence of other associated congenital anomalies like Down syndrome, congenital heart disease, omphalocele and pectus carinatum.[1-4] Transverse colon and omentum are the most common contents. Most of the patients are asymptomatic and this leads to the delayed diagnosis. Rarely patients may present with acute intestinal obstruction and perforation. MH presenting as obstructive jaundice is extremely rare and two published reports were found on literature search.[5] The cause of obstructive jaundice was traction and rotation of the distal common bile duct due to the herniation of the bowel along with the second part of duodenum in the index case.

## Footnotes

**Source of Support:** Nil

**Conflict of Interest:** None declared

